# Degradation of Glyphosate in Water by Electro-Oxidation on Magneli Phase: Application to a Nanofiltration Concentrate

**DOI:** 10.3390/molecules30153153

**Published:** 2025-07-28

**Authors:** Wiyao Maturin Awesso, Ibrahim Tchakala, Sophie Tingry, Geoffroy Lesage, Julie Mendret, Akpénè Amenuvevega Dougna, Eddy Petit, Valérie Bonniol, Mande Seyf-Laye Alfa-Sika, Marc Cretin

**Affiliations:** 1 Institut Européen des Membranes (IEM), UMR 5635, University of Montpellier, ENSCM, CNRS, 34090 Montpellier, France; wiyao-maturin.awesso@umontpellier.fr (W.M.A.); sophie.tingry@umontpellier.fr (S.T.); geoffroy.lesage@umontpellier.fr (G.L.); julie.mendret@umontpellier.fr (J.M.); eddy.petit@umontpellier.fr (E.P.); valerie.bonniol@umontpellier.fr (V.B.); 2 Laboratory of Water Resources and Environmental Engineering, Faculty of Sciences and Technology, University of Kara, Kara BP 404, Togo; itchakala@univ-lome.tg (I.T.); adougna@yahoo.fr (A.A.D.); 3 Applied Hydrology and Environmental Laboratory (Formerly Water Chemistry Laboratory), University of Lomé, Lomé BP 1515, Togo; 4 Beijing Key Laboratory of Water Resources Environmental Engineering, China University of Geosciences (Beijing), Beijing 100083, China

**Keywords:** glyphosate, electrochemical processes, advanced oxidation, Ti_4_O_7_, mineralization

## Abstract

This study evaluates the efficiency of sub-stoichiometric Ti_4_O_7_ titanium oxide anodes for the electrochemical degradation of glyphosate, a persistent herbicide classified as a probable carcinogen by the World Health Organization. After optimizing the process operating parameters (pH and current density), the mineralization efficiency and fate of degradation by-products of the treated solution were determined using a total organic carbon (TOC) analyzer and HPLC/MS, respectively. The results showed that at pH = 3, glyphosate degradation and mineralization are enhanced by the increased generation of hydroxyl radicals (^●^OH) at the anode surface. A current density of 14 ${\mathrm{mA}\;\mathrm{cm}}^{-2}$ enables complete glyphosate removal with 77.8% mineralization. Compared with boron-doped diamond (BDD), ${\mathrm{Ti}}_{4}O_{7}$ shows close performance for treatment of a concentrated glyphosate solution (0.41 mM), obtained after nanofiltration of a synthetic ionic solution (0.1 mM glyphosate), carried out using an NF-270 membrane at a conversion rate (Y) of 80%. At 10${\;\mathrm{mA}\;\mathrm{cm}}^{-2}$ for 8 h, ${\mathrm{Ti}}_{4}O_{7}$ achieved 81.3% mineralization with an energy consumption of 6.09 ${\mathrm{kWh}\;g}^{-1}$ TOC, compared with 90.5% for BDD at 5.48 ${\mathrm{kWh}\;g}^{-1}$ TOC. Despite a slight yield gap, ${\mathrm{Ti}}_{4}O_{7}$ demonstrates notable efficiency under demanding conditions, suggesting its potential as a cost-effective alternative to BDD for glyphosate electro-oxidation.

## 1. Introduction

The efficient role of glyphosate as an inhibitor of the 5-enolpyruvylshikimate-3-phosphate synthase (EPSPS) enzyme of the shikimate pathway, present in a very large number of plant species, makes it a unique herbicide [1,2,3]. Quantities of glyphosate-based herbicides, under the trade name Roundup^®^, have increased dramatically since 1974, and in 2014 nearly 0.53 kg/ha were sprayed on all cropland worldwide [4]. Although the actual impacts of this systemic, non-selective herbicide, as well as its fate and degradation mechanisms, are still controversial [5,6], glyphosate is causing concern, and some European countries have already banned its application on food crops. In 2015, the World Health Organization’s International Agency for Research on Cancer placed glyphosate in Group 2A of substances probably carcinogenic to humans. In addition, its accumulation and that of its metabolites also pose potential toxicological problems of long-term effects at very low doses on the health of aquatic organisms [7,8]. Consequently, the elimination of glyphosate from water resources has attracted scientific interest.

Contamination of glyphosate, its coadjuvants, and its degradation metabolites (such as aminomethylphosphonic acid or AMPA, sarcosine, and glycine) in surface water and groundwater can occur through surface runoff, direct herbicide spraying, inappropriate application practices, and disposal of herbicide wastes [6,9]. Those toxic molecules can persist in water for several months due to their relatively long half-lives (t_1/2 glyphosate_, 45 to 60 days; t_1/2 AMPA_, 76 to 240 days) [10,11].

In Togo, glyphosate has been integrated into agricultural practices; however, in the face of environmental and health concerns, the Togolese government has banned its import, marketing, and use since December 2019. However, increased use of glyphosate has been recorded in coffee and cocoa plantations in neighboring countries such as Ghana [12]. Due to its porous borders, Togo faces a worrying situation, as it constitutes a market for the sale, use, and/or transit of various products with often uncertain characteristics. A study from 2021 has revealed glyphosate contamination of Togolese soils and crops [13], suggesting possible contamination of drinking water resources. In the USA, where glyphosate consumption accounts for 19% of worldwide use, the presence of this herbicide and its main metabolite, AMPA, has been reported in most stream and river samples, with concentrations up to 430 ${µg\;L}^{-1}$ [14]. The persistence and widespread presence of these molecules in different environments (water, soil, and air) are influenced by various environmental factors such as temperature, pH, and the presence of organic matter [15].

A variety of processes can be used to remove glyphosate from natural water and wastewater, including biological, physicochemical and advanced oxidation processes (AOP). These different methods have advantages and limitations in terms of efficiency, cost, feasibility, and environmental impacts. Physicochemical processes (such as coagulation, adsorption, and reverse osmosis), although non-destructive, do not result in total destruction of the organic chemicals and require costly post-treatment [16,17]. On the other hand, biological oxidation based on enzymatic reactions is a slow process that can generate toxic by-products if operating conditions are not controlled [18,19]. AOPs have shown their effectiveness in the treatment of toxic and non-biodegradable organic pollutants [20,21,22]. These processes generate in situ hydroxyl radicals (^●^OH) [23], which are powerful oxidants (E° = 2.80 V/ESH) that can easily oxidize any organic or organometallic pollutant up to the final oxidation stage ($CO_{2}$ and $H_{2}O$). In particular, anodic electro-oxidation of water is a process that generates ^●^OH in situ at an electrode surface, without adding reagents. The main limitations of this technology concern the electrical supply costs, organic matter quenching, and electrode lifetime.

To efficiently produce ^●^OH by electrochemical oxidation of water and consequently high rates of oxidation and mineralization of pollutants [24], the choice of the anode material is important to consider. Electro-oxidation is preferably performed on electrodes (M) with high oxygen overpotential according to Equation (1) to promote the production of radicals.

(1) $$M+H_{2}O\;\to \;M\;{(}^{●}\mathrm{OH})+H^{+}+e^{-}$$

These electrodes must also exhibit stability in saline electrolytic media, a long lifetime, and good cost-effectiveness from environmentally friendly materials. The performance of electrochemical oxidation is influenced by operational parameters like the pH of the pollutant’s solution, the initial pollutant concentration, the electrolyte nature, and the current density.

Several anode materials have been employed. Boron-doped diamond (BDD) is well known material to be efficient for anodic oxidation of glyphosate at high concentration (100 × $10^{3}$–360 × $10^{3}$ ${µg\;L}^{-1}$) using high current density (10–100 ${\mathrm{mA}\;\mathrm{cm}}^{-2}$), both in laboratory and pre-pilot scales [25]. BDD electrodes are generally prepared by high-pressure sintering (5 GPa) and at temperatures above 1500 °C [26], or by chemical vapor deposition (CVD) on a conductive substrate (such as silicon, niobium or, more recently, titanium) [27,28]. However, the high cost of the material and actual scarcity of suitable substrates limit its large-scale application. Dimensionally stable mixed oxide anodes (DSA) have also been studied for glyphosate removal, but only 32% of glyphosate and 24% of total organic carbon (TOC) were removed for the best electrode $\mathrm{Ti}/{\mathrm{Ir}}_{30}{\mathrm{Sn}}_{0.70}O_{2}$ at 50 ${\mathrm{mA}\;\mathrm{cm}}^{-2}$ after 4 h [29]. Recently, Magneli phase ${\mathrm{TiO}}_{2}$ suboxides have been proposed as an alternative material for anodic oxidation process [30,31]. These types of phases, generally indicated as ${\mathrm{Ti}}_{n}O_{2n-1}$ (4 ≤ *n* ≤ 10), can be produced by the carbo-reduction of ${\mathrm{TiO}}_{2}$ at a high temperature of over 900 °C. Among a series of suboxides, ${\mathrm{Ti}}_{4}O_{7}$, is the most conductive and has shown great potential as an electrode for the generation of ^●^OH radicals [32]. Conventional ${\mathrm{Ti}}_{4}O_{7}$ plate electrodes have been studied to treat different types of pharmaceutical pollutants, such as paracetamol and amoxicillin, or per- and polyfluoroalkyl substances [31,33,34,35]. These electrodes could bridge the gap between efficiency and cost. However, their application to glyphosate degradation and mineralization by electro-oxidation, particularly in conjunction with pre-treatment processes such as nanofiltration (NF), an interesting alternative to reverse osmosis, has yet to be fully explored. Nevertheless, a study has been carried out on the use of advanced electrochemical oxidation processes with innovative electrode materials for mineralization and improved biodegradability of landfill leachate nanofiltration concentrate [36], demonstrating the potential of this approach for other organic micropollutants such as glyphosate. By concentrating pollutants in a retentate, NF reduces the volume to be treated and potentially increases the efficiency of electro-oxidation, a crucial synergy for countries with limited resources.

The electrochemical oxidation process depends on several experimental parameters whose optimization is essential for efficient degradation. As the efficiency of this process relies on the production of ^●^OH produced from water electrochemical oxidation at the anode surface [37], the nature of the anode material, the applied current, the initial concentration of the pollutant(s), and the pH of the medium are operational parameters to consider. The objective of this work is therefore to optimize the operating parameters of the process focusing on the pH and current density, and to evaluate the mineralization efficiency and degradation by-products of the treated glyphosate solution by determining the total organic carbon decomposition. A possible reaction mechanism for the electrochemical mineralization of glyphosate is proposed by analyzing and quantifying the degradation by-products, short-chain carboxylic acids and inorganic ions released. Finally, a comparison of the performance of ${\mathrm{Ti}}_{4}O_{7}$ with that of BDD is carried out in the case simulating the treatment of a retentate obtained after nanofiltration on a synthetic ionic solution polluted with glyphosate. These results could contribute to the improvement, or even development, of treatments for glyphosate-contaminated water, and reduce the impact of glyphosate on the environment and human health.

## 2. Results and Discussion

### 2.1. Effect of pH on Glyphosate Degradation and Mineralization

**Figure 1:**
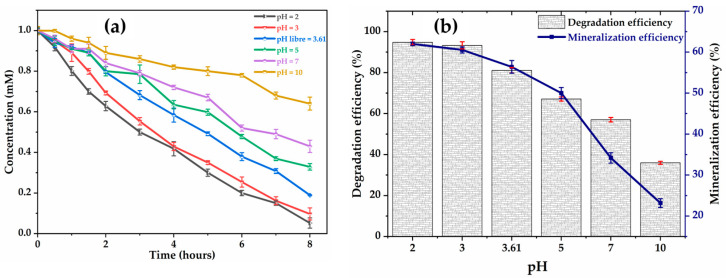
Effect of pH on: (**a**) glyphosate degradation; (**b**) mineralization. [Glyphosate] = 1 mM; [Na_2_SO_4_] = 50 mM; V = 200 mL; J = 6 ${\mathrm{mA}\;\mathrm{cm}}^{-2}$; Ѳ = 20 °C.

Electrochemical oxidation is strongly influenced by the pH of the medium, which modulates both the generation of ^●^OH, their reactivity, and the adsorption of pollutants at the electrode surface. The effect of initial pH on the rate of degradation and mineralization of glyphosate was therefore experimentally examined at the current density of 6 ${\mathrm{mA}\;\mathrm{cm}}^{-2}$ applied to the ${\mathrm{Ti}}_{4}O_{7}$ anode. The tests compared six pH conditions varying between 2 and 10. As demonstrated in Figure 1a, acidic conditions favor the degradation and mineralization of glyphosate. After 8 h of treatment (Figure 1b), the highest removal rates of glyphosate 94.80%; 93.30% and TOC 62.00%; 60.53% are recorded at pH = 2 and 3, respectively. At pH = 3.6 and pH = 5, the degradation efficiency decreases by 14% and 28%, respectively. Similarly, the values of the TOC removal are lower: 56.4% at pH 3.61 and 50% at pH = 5. As suggested by S. Aquino Neto et al. [29], low pH conditions decrease the undesirable oxygen release reaction forming $O_{2}$ at the electrode surface, rather favoring the formation of ^●^OH through water hydrolysis (Equation (2)) and, thus, the oxidation of organic compounds.

(2) H2O→OH●+H++e−

The four ionization constants of glyphosate, pK_a1_ = 0.8 (1st phosphonic), pK_a2_ = 2.6 (carboxylate), pK_a3_ = 5.6 (2nd phosphonic), and pK_a4_ = 10.6 (amine) [38] explain that the glyphosate molecule is characterized by an electric charge dependent on the pH of the solution. In an acidic medium, glyphosate adopts a neutral or slightly positive charge, which facilitates its adsorption on the anode surface (generally negatively charged), optimizing its contact with the ^●^OH and accelerating its degradation. This phenomenon explains the low degradation (36%) and mineralization (23.2%) rates at pH = 10, i.e., a decrease of 37%) in mineralization compared to pH = 3. These observations are consistent with the work of Aquino Neto et al. (2009), who demonstrated a direct correlation between acidic pH and increased ^●^OH production on dimensionally stable anode electrodes ($\mathrm{Ti}/{\mathrm{Ir}}_{0.30}{\mathrm{Sn}}_{0.70}O_{2}\;)$ when treating glyphosate (6 mM) by electrochemical oxidation at a current density of $50{\;\mathrm{mA}\;\mathrm{cm}}^{-2}$ [29]. After 4 h of treatment, a decrease in glyphosate degradation efficiency was observed along with an increase in pH as follows: 32% at pH = 2, 26% at pH = 5, and 15% at pH = 11 [29]. Furthermore, studies by Nam Tran et al. (2017) reveal that pH plays a critical role in the efficiency of electrochemical degradation of glyphosate (0.1 mM), as it was observed a decrease in glyphosate adsorption on metal oxides ($\mathrm{Ti}/{\mathrm{PbO}}_{2})$ at pH > 5 and current intensity of 5 A [39]. Therefore, for further study in this work, the glyphosate solution was set at an initial pH = 3.

### 2.2. Effect of Current Density on Glyphosate Degradation

**Figure 2:**
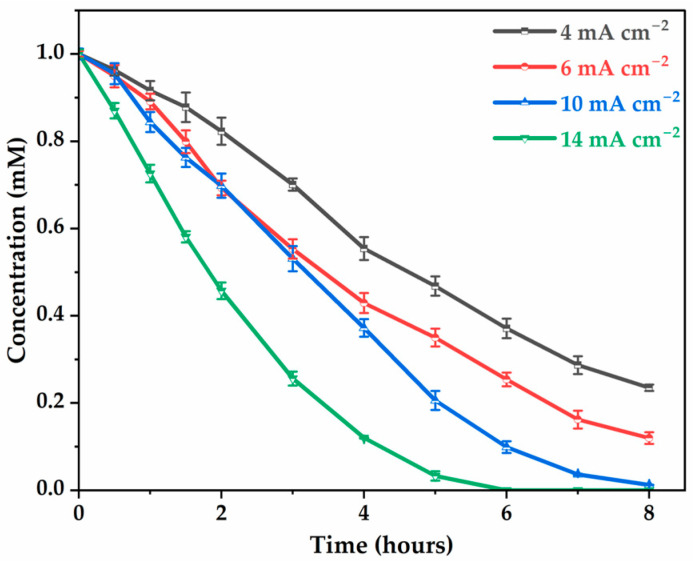
Effect of current density on glyphosate degradation. [Glyphosate] = 1 mM; [${\mathrm{Na}}_{2}{\mathrm{SO}}_{4}$] = 50 mM; V = 200 mL; pH = 3; Ѳ = 20 °C.

We next investigate the effect of current density on glyphosate degradation by measuring the residual glyphosate concentration after 8 h at various current densities. As observed in Figure 2, the percentages of degradation efficiency are 76.5%, 90.4%, 98.8%, and 100% for current densities of 4 ${\mathrm{mA}\;\mathrm{cm}}^{-2}$, 6 ${\mathrm{mA}\;\mathrm{cm}}^{-2}$, 10 ${\mathrm{mA}\;\mathrm{cm}}^{-2}$, and 14 ${\mathrm{mA}\;\mathrm{cm}}^{-2}$, respectively, indicating that the higher the current density, the more abundant the highly reactive ^●^OH that can be continuously generated at the anode surface [40,41], resulting in faster degradation amount and kinetics. Between 10 ${\mathrm{mA}\;\mathrm{cm}}^{-2}$ and 14 ${\mathrm{mA}\;\mathrm{cm}}^{-2}$, of glyphosate to the electrode surface, showing a decrease in degradation tendency.

Degradation kinetics were assumed to follow pseudo-first-order decay kinetics, with correlation coefficients ranging from 0.90 to 0.98 predicting a linear variation with respect to time (*t*) of:

(3) $$-\ln(\frac{C}{C_{0}})=k_{\mathrm{app}}\cdot t$$

where *C* is the concentration of glyphosate at time *t*, $C_{0}$ is the initial concentration of glyphosate, $k_{\mathrm{app}}$ is the pseudo-first-order degradation rate constant. The rate constant increased progressively with current density (Table 1), with values of 18.6 ± 0.7, 26.4 ± 1.1, 51.6 ± 5.7, 65.3 ± 6.2 (10^2^ min^−1^) corresponding to a current density of 4 to 10 ${\mathrm{mA}\;\mathrm{cm}}^{-2}$. Pseudo-first-order kinetics suggest that glyphosate degradation is responsible for a constant concentration of hydroxyl radicals, which were continuously generated at the anode surface [31,37]. The results confirm that the higher the current density, the more abundant the production of highly reactive ^●^OH, resulting in faster degradation of glyphosate molecules in terms of kinetic rate.

### 2.3. Effect of Current Density on Glyphosate Mineralization

Complete degradation of glyphosate does not necessarily mean the elimination of the pollution problem if the resulting “reaction intermediates” are more toxic than the parent molecule, as is the case with AMPA [42]. For this reason, complete mineralization means complete destruction of the pollutant into ${\mathrm{CO}}_{2}$ and $H_{2}O$ in solution and detoxification of the solution. The potential of ${\mathrm{Ti}}_{4}O_{7}$ as a suitable anode for the electrochemical oxidation of glyphosate was evaluated by studying the mineralization of a 1 mM glyphosate solution (corresponding to 39.91 ${\mathrm{mg}\;L}^{-1}$, initial TOC) at the applied current density ranging from 4 to 14 ${\mathrm{mA}\;\mathrm{cm}}^{-2}$.

**Figure 3:**
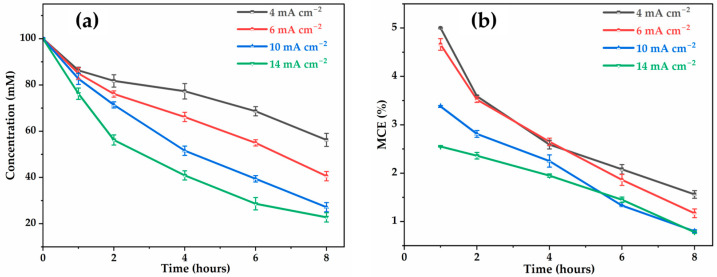
Effect of applied current on: (**a**) TOC removal; (**b**) mineralization current efficiency. [Glyphosate] = 1 mM; [${\mathrm{Na}}_{2}{\mathrm{SO}}_{4}$] = 50 mM; V = 200 mL; pH = 3; Ѳ = 20 °C.

Figure 3a represents the TOC decay as a function of electrolysis time at different applied currents. The percentage of oxidizable carbon (mineralized C-${\mathrm{CO}}_{2}$) for total carbon in the final solution after 8 h at 4, 6, 10, and 14${\;\mathrm{mA}\;\mathrm{cm}}^{-2}$ are 43.8%, 59.4%, 72.9%, and 77.8%, respectively. This confirms that as the current density increased, the mineralization efficiency also increased. This behavior is consistent with the generation of a large amount of M(^●^OH) from water oxidation (Equation (1)), leading to rapid oxidation of glyphosate and its intermediates. However, poor mineralization is observed at the lowest current densities (4 and 6 ${\mathrm{mA}\;\mathrm{cm}}^{-2}$), due to insufficient anodic potential (lower than the water discharge potential), limiting the production of ^●^OH [32,43]. The evolution of mineralization current efficiency (MCE, Equation (9)) as a function of time, illustrated in Figure 3b, shows a significant decrease in MCE as the current density increases. At 4 ${\mathrm{mA}\;\mathrm{cm}}^{-2}$ the MCE reaches around 5% at 1 h of electrolysis before decreasing to 1.7% after 8 h, while at 14 ${\mathrm{mA}\;\mathrm{cm}}^{-2}$, the MCE is only 2.6% and drops rapidly to a value below 1% after 8 h. This decline reflects the increasingly inefficient use of current for glyphosate mineralization, to the benefit of undesirable secondary reactions (e.g., oxygen evolution). Therefore, although increasing current density can accelerate certain degradation steps, it does not translate into an improvement in overall process efficiency. On the contrary, high current densities could lead to a loss of selectivity in the electrochemical process, higher energy consumption, as well as an increased risk of the ${\mathrm{Ti}}_{4}O_{7}$ electrode deterioration [44,45]. These penalizing effects make it inadvisable to use densities higher than 14 ${\mathrm{mA}\;\mathrm{cm}}^{-2}$. Therefore, to ensure good energy efficiency and system durability, it is preferable to work at lower densities, particularly around 4 to 10 ${\mathrm{mA}\;\mathrm{cm}}^{-2}$, which offer a better compromise between mineralization performance and power consumption.

**Figure 4:**
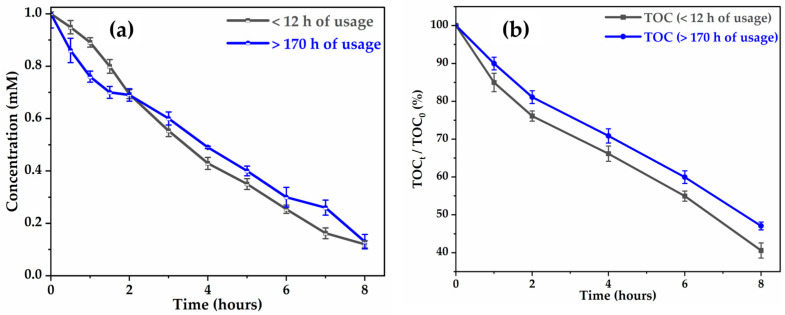
Stability of activity of ${\mathrm{Ti}}_{4}O_{7}$ with usage time for degradation (**a**) and mineralization (**b**).

The stability of the activity of ${\mathrm{Ti}}_{4}O_{7}$ was evaluated by comparing the performance of glyphosate degradation and mineralization after 12 h and after 170 h of use in electro-oxidation at 6 ${\mathrm{mA}\;\mathrm{cm}}^{-2}$. After 170 h, mineralization efficiency decreases by around 6.5% (Figure 4b), while the decrease in glyphosate degradation is negligible (Figure 4a) after 8 h of electrolysis. This slight loss of activity could be explained by the progressive oxidation of the $T_{i}^{3+}$ (from ${\mathrm{Ti}}_{4}O_{7}$) in $T_{i}^{4+}$ leading to the formation of a dense film of ${\mathrm{TiO}}_{2}$ (low-conductivity rutile phase) of a few nanometers on the anode surface, which prevents hydroxyl radicals from accessing the active sites [46].

### 2.4. Evaluation of Energy Consumption During the Degradation and Mineralization of Glyphosate

**Figure 5:**
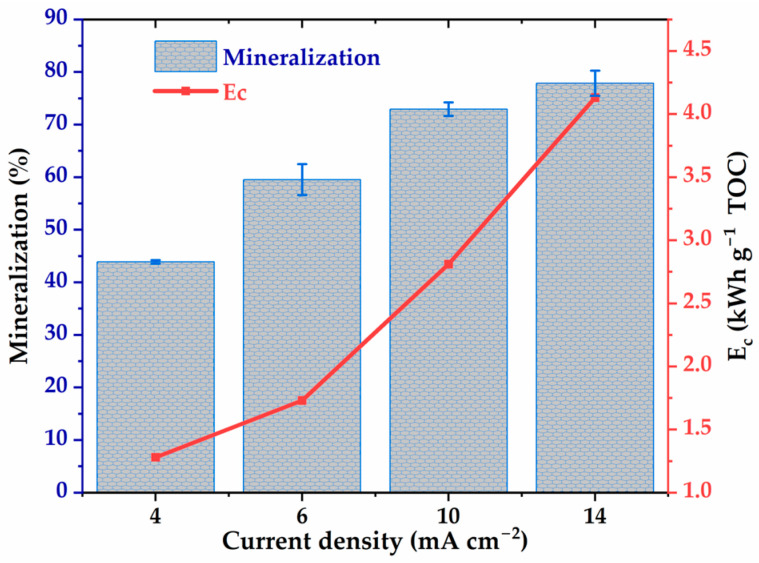
The energy consumption and TOC removal efficiency as a function of the density of the electrolysis current. [Glyphosate] = 1 mM; [${\mathrm{Na}}_{2}{\mathrm{SO}}_{4}$] = 50 mM; V = 200 mL; pH = 3; Ѳ = 20 °C.

Current density is a key parameter in electro-oxidation, directly influencing the generation of oxidizing species at the anode as well as the energy consumption of the process. In this study, the effect of current density on glyphosate mineralization and specific energy (Ec) was evaluated in the range of 4 to 14 ${\mathrm{mA}\;\mathrm{cm}}^{-2}$, as shown in Figure 5. The results show a significant increase in the TOC mineralization rate with current density, from 43% to 77.8% between 4 and 14 ${\mathrm{mA}\;\mathrm{cm}}^{-2}$. This improvement is attributed to an increased production of hydroxyl radicals at the ${\mathrm{Ti}}_{4}O_{7}$ anode at higher current densities, promoting the degradation of organic compounds. However, the gain becomes marginal beyond 10 ${\mathrm{mA}\;\mathrm{cm}}^{-2}$, suggesting an efficiency plateau, likely as nothing before. At the same time, the specific energy increases markedly, reaching 4.35 ${\mathrm{kWh}\;g}^{-1}$ TOC at 14 ${\mathrm{mA}\;\mathrm{cm}}^{-2}$. According to Equation (11), this observation can be explained by the evolution of the cell voltage from 4.8 V to 5.7 V, 7.8 V, and up to 8.7 V by varying the current density by 4, 6, 10, and 14 ${\mathrm{mA}\;\mathrm{cm}}^{-2}$, respectively. Note that for each current density value, the voltage fluctuations observed during the electrolysis time (8 h) were less than 10%. This result reflects a reduction in energy efficiency at high current density, as energy costs increase faster than mineralization efficiency. Therefore, an intermediate density, between 4 and 10 ${\mathrm{mA}\;\mathrm{cm}}^{-2}$, appears to be an optimal compromise, allowing for a good mineralization rate while limiting energy consumption.

These observations highlight the importance of fine-tuning electrochemical parameters to ensure both treatment efficiency and energy viability. Future work could explore coupling the process with complementary techniques (photo-electro-Fenton, biological pretreatment, etc.) to increase overall efficiency while reducing energy consumption.

### 2.5. Identification of Intermediate and Proposed Degradation Pathways

The degradation of pesticides by AOP generates, through a series of chemical reactions, various by-products and intermediate compounds. Their identification and monitoring are essential to assess the efficiency of the process and its ecological impact. Concerning glyphosate, its degradation results mainly from the action of ^●^OH capable of breaking C-N and C-P bonds. This fragmentation results in intermediates such as AMPA (persistent and potentially toxic metabolite) and sarcosine, then to final products such as nitrate, ammonium and phosphate ions, as well as carbon dioxide and water [47] (Equation (4)).

(4) 2C3H8NO5P +38OH●→6 CO2 +25 H2O+NO3−+NH4++2 PO43−

**Figure 6:**
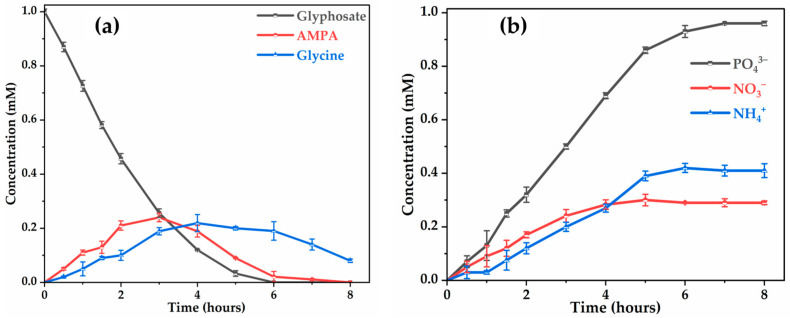
Evolution of the concentration of: (**a**) glyphosate, glycine and AMPA with time; and (**b**) phosphate, ammonium and nitrate ions. [Glyphosate] = 1 mM; [${\mathrm{Na}}_{2}{\mathrm{SO}}_{4}$] = 50 mM; [$K_{2}{\mathrm{SO}}_{4}$] = 50 mM (for ${\mathrm{NH}}_{4}^{+})$; V = 200 mL; pH = 3; J = 14 ${\mathrm{mA}\;\mathrm{cm}}^{-2}$; Ѳ = 20 °C.

**Figure 7:**
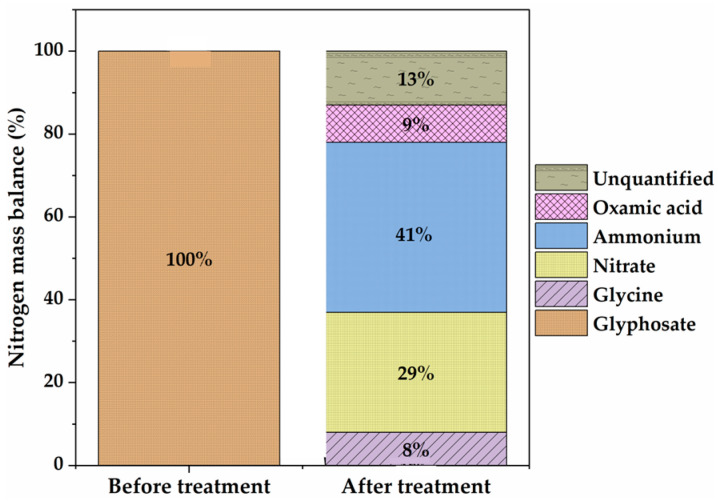
Nitrogen mass balance before and after electro-oxidation. [Glyphosate] = 1 mM; [${\mathrm{Na}}_{2}{\mathrm{SO}}_{4}$] = 50 mM; [$K_{2}{\mathrm{SO}}_{4}$] = 50 mM (for ${\mathrm{NH}}_{4}^{+})$; V = 200 mL; pH = 3; J = 14 ${\mathrm{mA}\;\mathrm{cm}}^{-2}$; Ѳ = 20 °C.

**Figure 8:**
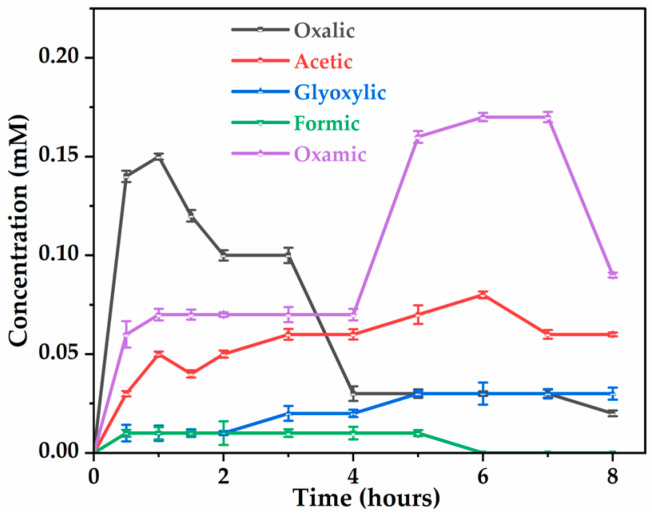
Evolution of carboxylic acids during glyphosate degradation. [Glyphosate] = 1 mM; [Na_2_SO_4_] = 50 mM; V = 200 mL; pH = 3; J = 14 ${\mathrm{mA}\;\mathrm{cm}}^{-2}$; Ѳ = 20 °C.

In our study, glycine and AMPA were the two dominant by-products detected, resulting from the attack of C-N and C-P bonds of glyphosate molecules by hydroxyl radicals (Figure 6a). The formation of AMPA and glycine during the first two hours of treatment appears to be at the same rate. However, the degradation of AMPA was more favorable than that of glycine since AMPA disappears after 8 h, while glycine remains the dominant by-product at the end of the treatment. The persistence of glycine can be explained by its slower transformation into ${\mathrm{CO}}_{2}$ and ${\mathrm{NH}}_{4}^{+}$/${\mathrm{NO}}_{3}^{-}$, its total degradation requiring several intermediates, such as the formation of oxalic acid (Figure 7). No trace of sarcosine (N-methylglycine) was detected during the treatment process, whereas sarcosine was detected by electro-oxidation of glyphosate herbicide at different DSA^®^ electrodes [29] and by removal of glyphosate by electrochemically assisted ${\mathrm{MnO}}_{2}$ oxidation process [48]. Lan et al. (2013) [48] also proposed a mechanism for glyphosate degradation by identifying glycine and sarcosine as the first intermediates that are then oxidized to oxamic acid and glycolic acid, both of which are ultimately transformed into acetic acid and ${\mathrm{NH}}_{4}^{+}$ and ${\mathrm{NO}}_{3}^{-}$. This discrepancy can be explained by the nature of electrode materials, influencing the reaction mechanisms. The absence of sarcosine detected suggests its rapid transformation into glycine under the electrochemical conditions applied with ${\mathrm{Ti}}_{4}O_{7}$ (Figure 8).

The presence of phosphate (${\mathrm{PO}}_{4}^{3-}$), ammonium (${\mathrm{NH}}_{4}^{+}$) and nitrate (${\mathrm{NO}}_{3}^{-})$ ions during glyphosate mineralization was also observed (Figure 6b). Obviously, the concentrations of these ions increase with the reaction time, indicating that the primary chemical structure of glyphosate was destroyed and ammonium was oxidized to nitrite and then to nitrate. The amount of ${\mathrm{NH}}_{4}^{+}$ and ${\mathrm{NO}}_{3}^{-}$ ions continuously accumulate in the treated solution for 8 h with the ${\mathrm{Ti}}_{4}O_{7}$ anode. Most of the released nitrogen atom was detected as ${\mathrm{NH}}_{4}^{+}$, with a significant proportion as ${\mathrm{NO}}_{3}^{-}$, which could be explained by the partial reduction in the small fraction of ${\mathrm{NO}}_{3}^{-}$ formed to ${\mathrm{NH}}_{4}^{+}$. The nitrite was not detected in the system, probably due to its quick oxidation to nitrate by ^●^OH [49]. In addition, ammonium (${\mathrm{NH}}_{4}^{+}$) and nitrate (${\mathrm{NO}}_{3}^{-})$ were found to accumulate from pesticides containing nitrogen atoms [50].

After 8 h of electrolysis, 0.41 mM ${\mathrm{NH}}_{4}^{+}$ and 0.29 mM ${\mathrm{NO}}_{3}^{-}$, representing 41% and 29% of the initial N atom in a glyphosate solution (1 mM N), respectively, were found in the final treated solution. Although organic P was completely recovered as ${\mathrm{PO}}_{4}^{3-}$, in the treated solutions, the amount of inorganic N was much lower than the initial total N content of the 1 mM glyphosate solution (i.e., 70%). The N mass balance is slightly deficient, which explains the relatively acceptable mineralizing power of the ${\mathrm{Ti}}_{4}O_{7}$ anode (9% of the N is present in the unmineralized oxamic acid) (Figure 7). The remaining 13% of undetected nitrogen may have been lost as volatile nitrogen compounds ($N_{x}O_{y}$) [51,52].

Focusing on the mineralization of glyphosate (Figure 3a), it was shown that only a partial reduction in TOC is achieved, while its primary toxic by-product, AMPA, is completely mineralized (Figure 6a), meaning that other intermediates (carboxylic acids) limit the mineralization process of herbicide. The analysis of the solution by ion exclusion chromatography treated at different electrolysis times showed the formation of several carboxylic acids, such as oxalic, acetic, oxamic, formic, and glyoxylic acids, resulting from the cleavage of intermediate by-products. The evolution of these carboxylic acids, shown in Figure 8, indicates low accumulation. Oxalic acid (0.02 mM) and oxamic acid (0.09 mM) are strongly involved in the glyphosate degradation pathway after 8 h of electrolysis, with oxamic acid appearing to be more stable over the long term. The accumulation of acetic acid suggests that it is a product of degradation; however, glyoxylic and formic acids are short-lived transient intermediates. The decrease in oxamic, oxalic, and formic acids after a peak may indicate their mineralization to ${\mathrm{CO}}_{2}$ and $H_{2}O$. It should be noted that the persistence of these carboxylic acids after 8 h of electrolysis may explain the percentages of residual TOC (Figure 3a) observed in the treated solution.

**Figure 9:**
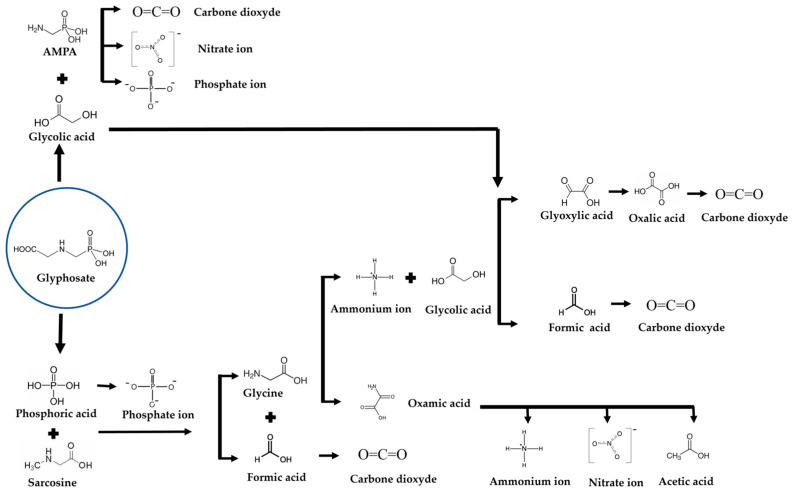
Proposed degradation pathway of glyphosate by electro-oxidation on ${\mathrm{Ti}}_{4}O_{7}$ Magneli phase.

In Figure 9, a reaction mechanism of glyphosate mineralization is proposed from the degradation intermediates, carboxylic acids, and inorganic compounds at the end of the mineralization reaction. The formation of by-products simultaneously indicates that the degradation reaction is carried out by cleavage of C-P, C-N, and C-C bonds (Equations (4)–(6)). Phosphate ions can be generated either directly from glyphosate via C-P bond cleavage or indirectly via AMPA formation and C-P bond cleavage of this by-product. The fact that phosphate ions and AMPA are detected simultaneously from the outset means that the indirect route does not predominate over the direct route. The increased concentration of acetic acid and oxalic acid during glyphosate degradation also pointed out the cleavage of the C-P bond [53].

Glyphosate degradation to AMPA (C-N bond cleavage):

(5) $$C_{3}H_{8}{\mathrm{NO}}_{5}P\;+\;2H_{2}O\;\to \;{\mathrm{CH}}_{6}{\mathrm{NPO}}_{3}\;+\;3{\mathrm{CO}}_{2}+\;6H^{+}\;+6e^{-}$$

AMPA degradation (C-P bond cleavage): total mineralization

(6) $${\mathrm{CH}}_{6}{\mathrm{NPO}}_{3}+6H_{2}O\;\to \;{\mathrm{CO}}_{2}+{\;\mathrm{NO}}_{3}^{-}+{\;\mathrm{PO}}_{4}^{3-}+18H^{+}\;+14e^{-}$$

Direct degradation of glyphosate (cleavage of the C-P bond): total mineralization (cf. Equation (4)).

### 2.6. Evolution of the Toxicity of Degradation By-Products

We further analyzed the evolution of the toxicity of degradation by-products from the determination of the % luminescence inhibition of *V. fischeri* bacteria as a function of the electrolysis time after 5 and 15 min of exposure during glyphosate mineralization with ${\mathrm{Ti}}_{4}O_{7}$ anode. Uncertainty analysis, quantified by the coefficient of variation (CV%), highlights the reliability of toxicity results. The coefficient of variation values (*n* = 5%) obtained were 12% for inhibition levels below 20%, 5% for inhibition levels between 20% and 70%, and 0% for inhibition levels above 70%, reflecting a rigorous statistical approach [54,55].

**Figure 10:**
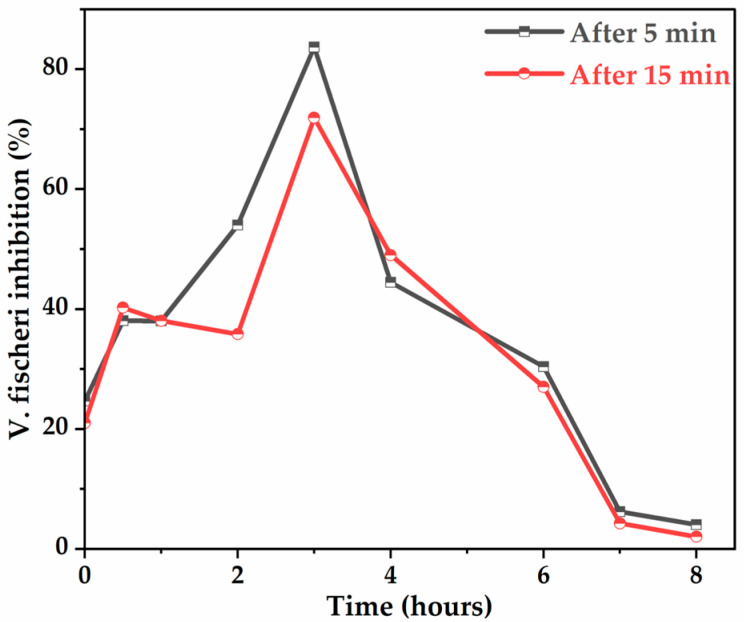
Evolution of the inhibition of the luminescence of V. fischeri bacteria (10% Microtox screening test) during glyphosate mineralization with ${\mathrm{Ti}}_{4}O_{7}$ anode. [Glyphosate] = 1 mM; [Na_2_SO_4_] = 50 mM; V = 200 mL; J = 14 ${\mathrm{mA}\;\mathrm{cm}}^{-2}$; Ѳ = 20 °C.

As shown in Figure 10, the glyphosate solution is initially slightly toxic to the bacteria, as the inhibition of luminescence is only 24.63%. The curves obtained are characterized by a significant increase in the % of luminescence inhibition during the first 2 h and reach a maximum of 83.67% (after 5 min of exposure) and 71.9% (after 15 min of exposure) around 3 h of electrolysis, due to the maximal concentration of AMPA, known to be toxic (Figure 6a), and other intermediates. After approximately 3 h of treatment, the curves indicate a rapid decrease in the % of luminescence inhibition to a lower value of 2% at 8 h of electrolysis, suggesting that the toxicity to bacteria of the treated glyphosate solutions gradually disappears, confirming degradation or elimination of toxic by-products.

## 3. Electro-Oxidation of Nanofiltration Retentate

Building on the satisfactory results obtained from the electrochemical degradation of glyphosate at an initial concentration of 1 mM, particularly in terms of mineralization efficiency and energy performance, it is now pertinent to extend this approach to the treatment of concentrates resulting from nanofiltration (NF).

Nanofiltration (NF) processes, although effective in concentrating pollutants such as glyphosate and its metabolite, AMPA, generate concentrated effluents, rich in recalcitrant compounds and salts, which cannot be discharged without risk to the environment, particularly due to the persistence of glyphosate (half-life of 45 to 315 days) and its chronic toxicity. The coupling of membrane separation with electrochemical treatment therefore represents a promising strategy to enhance cost effectiveness of treatment while minimizing the environmental impact of such concentrates. The purpose of this work was to evaluate the performance of the ${\mathrm{Ti}}_{4}O_{7}$ anode in the treatment of the nanofiltration retentate of a synthetic ionic solution by anodic electro-oxidation, in comparison with experiments conducted with a BDD electrode (2 × 4 cm × 4 cm). By combining the two processes, the treatment chain could be optimized, as NF reduces the volume of water to be treated, while EO targets refractory compounds.

An NF-270 polyamide membrane from DOW Filmtec was selected in this study for its ability to combine high organic rejection, superior permeability, and low energy consumption [56]. This membrane is considered a “fine” NF membrane with a cutoff of 200 Da, which seems appropriate for glyphosate retention (PM ≈ 169 Da), as demonstrated by the study of Kimura et al. [57] that showed the better performance of polyamide membranes in terms of rejection of certain pharmaceuticals and endocrine disruptors compared with cellulose acetate membranes. In addition, NF-270 offers a good “retention-flow” compromise with permeabilities between 12 and 15 ${L\;h}^{-1}{\;m}^{-2}{\;\mathrm{bar}}^{-1}$. This compromise is explained by the fact that the membrane has both a low pore size (around 0.40 nm compared with 0.30 nm for NF-90 according to the literature) and a high pore number (1.46 × $10^{16}$ $m^{-2}$ compared to 0.22 × $10^{16}$ $m^{-2}$ for DL membrane).

Table 2 shows the ionic composition of the feed solution, permeate, and retentate obtained after 1 h of filtration at a transmembrane pressure (TMP) of 10 bar. The experiment was carried out with an applied flux of 143 LMH, corresponding to a volume concentration factor (VCF) of 4.3, indicating that the initial volume was reduced by a factor greater than 4 in the retentate.

**Figure 11:**
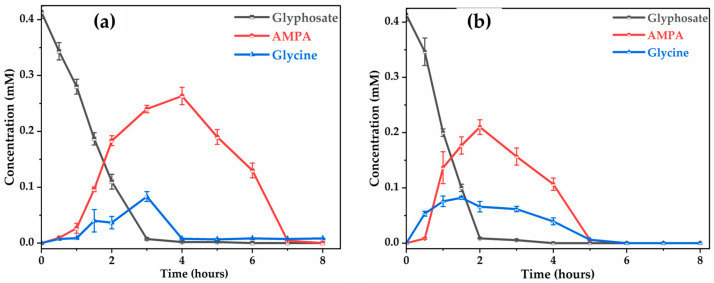
Evolution of the concentration of glyphosate, glycine and AMPA concentration with time on: (**a**) ${\mathrm{Ti}}_{4}O_{7}$; (**b**) BDD. [Glyphosate] = 72.3 mg $L^{-1}$ (0.41 mM); V = 200 mL; J = 10 ${\mathrm{mA}\;\mathrm{cm}}^{-2}$; pH = 8.45; Ѳ = 20 °C.

Comparison of the electrochemical performance of ${\mathrm{Ti}}_{4}O_{7}$ and BDD for the electro-oxidation of glyphosate in synthetic solution (Figure 11) shows differences in terms of degradation kinetics, formation of intermediates, and mineralization capacity. On the ${\mathrm{Ti}}_{4}O_{7}$ electrode (Figure 11a), glyphosate degradation is progressive, reaching almost complete disappearance after 3 h of treatment. On the other hand, with the BDD electrode (Figure 11b), degradation is faster, with almost complete elimination of glyphosate after 2 h.

Degradation of the by-products, notably AMPA and glycine, reaches a higher maximum concentration and persists longer with the ${\mathrm{Ti}}_{4}O_{7}$. The peak AMPA concentration is observed at around 0.28 mM at 3 h on ${\mathrm{Ti}}_{4}O_{7}$ (Figure 11a), compared with 0.22 mM at 2 h on BDD (Figure 11b), showing slower partial oxidation with ${\mathrm{Ti}}_{4}O_{7}$. Furthermore, AMPA is no longer detectable at 7 h in the case of ${\mathrm{Ti}}_{4}O_{7}$ whereas it is eliminated at 5 h with BDD. The efficiency of the ${\mathrm{Ti}}_{4}O_{7}$ anode proved superior to that of the BDD electrode, with total glycine reduction achieved in 4 h, one hour less than the time required with BDD.

These observations confirm that the degradation efficiency of glyphosate on the ${\mathrm{Ti}}_{4}O_{7}$ anode is close to that of BDD anode. The difference could be explained by the better efficiency of the BDD electrode to promote the formation of free hydroxyl radicals thanks to its higher water decomposition potential. As pointed by Gherardini et al. [58], the ^●^OH formed on the surface of the BDD remains essentially free (physisorption), attacking organic compounds directly, whereas they are chemisorbed (therefore less mobile) on the surface of ${\mathrm{Ti}}_{4}O_{7}$ where they react more slowly with organic compounds.

With regard to the evolution of carboxylic acids, a notable distinction was observed concerning the evolution of formic acid, a common intermediate in glyphosate degradation [59]. On the ${\mathrm{Ti}}_{4}O_{7}$ anode, formic acid was almost eliminated after 7 h, whereas it persisted in trace amounts (0.012 mM) with BDD, even after this time. Interestingly, all acids were effectively mineralized during electrolysis on both electrodes, with slightly faster degradation kinetics in the case of the BDD anode compared to the ${\mathrm{Ti}}_{4}O_{7}$ anode.

**Figure 12:**
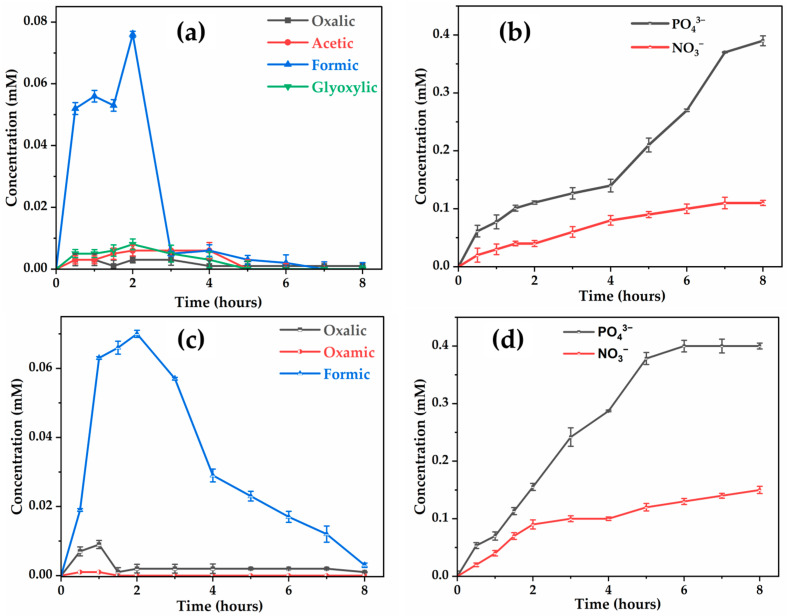
Evolution of carboxylic acids and ions concentrations on: ${\mathrm{Ti}}_{4}O_{7}$ (**a**,**b**); BDD (**c**,**d**) during EO of retentate. [Glyphosate] = 72.3 mg $L^{-1}$ (0.41 mM); V = 200 mL; J = 10 ${\mathrm{mA}\;\mathrm{cm}}^{-2}$; pH = 8.45; Ѳ = 20 °C.

During electrolysis of the nanofiltration concentrate containing 0.41 mM glyphosate at 10 ${\mathrm{mA}\;\mathrm{cm}}^{-2}$, the organic nitrogen released by the breaking of covalent bonds was mainly found in the form of ${\mathrm{NH}}_{4}^{+}$ and ${\mathrm{NO}}_{3}^{-}$ without detection of ${\mathrm{NO}}_{2}^{-}$, as shown by ion chromatography. These species accumulate over the 8 h of treatment, whether with the ${\mathrm{Ti}}_{4}O_{7}$ anode (Figure 12b) or BDD (Figure 12d), the latter showing slightly better mineralization efficiency. Indeed, 0.15 mM ${\mathrm{NO}}_{3}^{-}$ (i.e., 37% of the initial nitrogen) were found with BDD versus 0.11 mM (27%) with ${\mathrm{Ti}}_{4}O_{7}$. Organic phosphorus was fully converted into inorganic phosphate (${\mathrm{PO}}_{4}^{3-}$) ions, but the complete quantification of inorganic nitrogen was limited due to interferences between ammonium (${\mathrm{NH}}_{4}^{+}$) and sodium $({\mathrm{Na}}^{+}$) ions present in the concentrate, which made their separation difficult during ion chromatography analysis. The mass balance was almost complete with BDD, while the deficit observed with ${\mathrm{Ti}}_{4}O_{7}$ could be explained by the formation of volatile nitrogen compounds (NxOy) or the persistence of unidentified organic nitrogen compounds during this study.

### 3.1. Comparison of Mineralization and Energy Consumption

**Figure 13:**
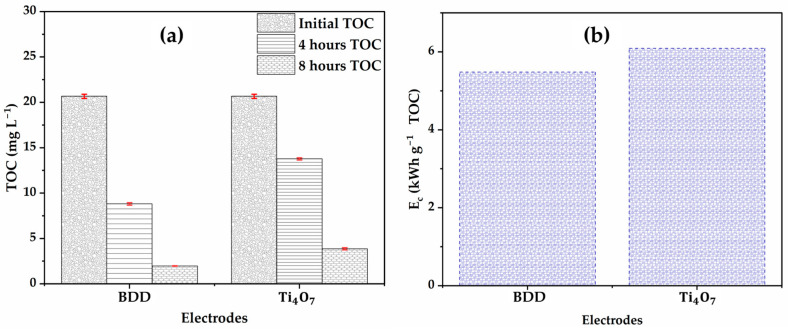
Evolution of: (**a**) TOC; (**b**) energy consumption during EO of the NF retentate. [Glyphosate] = 72.3 mg $L^{-1}$ (0.41 mM); V = 200 mL; J = 10 ${\mathrm{mA}\;\mathrm{cm}}^{-2}$; Ѳ = 20 °C.

Electrical energy consumption (EC) is a key parameter when scaling up a process for large-scale application in wastewater treatment. This parameter makes it possible to estimate the profitability of the process in environmental applications. In terms of glyphosate mineralization, the ${\mathrm{Ti}}_{4}O_{7}$ anode demonstrates an efficiency only 10% lower than that of the BDD (Figure 13a). After 8 h of treatment, the TOC concentration falls from 20.66 mg $L^{-1}$ to 1.96 mg $L^{-1}$ (90.5% mineralization) with the BDD, while it remains at nearly 3.86 mg $L^{-1}$ (81.3% mineralization) with the ${\mathrm{Ti}}_{4}O_{7}$. These results are in agreement with the observed faster degradation on the BDD anode than the ${\mathrm{Ti}}_{4}O_{7}$ anode (Figure 11) and the efficiency of BDD to produce unabsorbed hydroxyl radicals (^●^OH) in solution.

In terms of specific energy consumption expressed in kWh $g^{-1}$ of organic matters (Figure 13b), the values obtained are similar, 6.09 kWh $g^{-1}$ TOC for ${\mathrm{Ti}}_{4}O_{7}$ compared to 5.48 kWh $g^{-1}$ TOC for BDD after 8 electrolysis cycles. This observation can be explained (i) by the higher cell voltage for electrolysis on ${\mathrm{Ti}}_{4}O_{7}$ compared to BDD, respectively, 8.7 V and 7.9 V and (ii) by the complexity of the matrix treated, in particular the high salt content of the nanofiltration concentrate, which probably limited the performance of the treatment unit at ${\mathrm{Ti}}_{4}O_{7}$ by increasing ohmic resistance or promoting the formation of persistent organic intermediates. Conversely, BDD, thanks to its strong oxidizing power and efficient generation of hydroxyl radicals, enables more complete degradation, thus reducing specific energy consumption [60]. Despite this slight variation, these results indicate that the two systems have comparable energy performances, suggesting that the ${\mathrm{Ti}}_{4}O_{7}$ is a viable alternative to BDD, particularly when other criteria such as the degradation rate of intermediates (e.g., glycine and formic acid) or material costs are taken into account.

### 3.2. Evolution of the Toxicity of Glyphosate-Contaminated Waters by Nanofiltration/Electro-Oxidation Coupling

**Figure 14:**
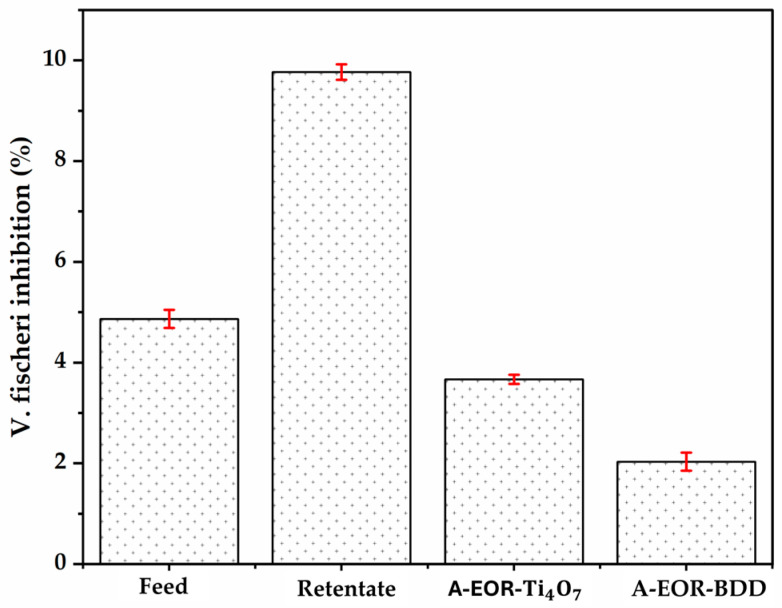
Evolution of acute toxicity (10% Microtox screening test, after 15 min of contact with *V. fischeri*) in feed, retentate, After Electro-oxidation (8 h) of the Retentate with ${\mathrm{Ti}}_{4}O_{7}$ (A-EOR-${\mathrm{Ti}}_{4}O_{7})$ and with BDD (A-EOR-BDD). Electro-oxidation condition: [Glyphosate] = 0.41 mM; V = 200 mL; J = 10 ${\mathrm{mA}\;\mathrm{cm}}^{-2}$.

Toxicity tests were carried out during (Figure 14). The initial synthetic solution containing glyphosate (0.1 mM) showed low inhibition (4.9%), consistent with the moderate toxicity of glyphosate at low concentrations for certain organisms such as *Vibrio fischeri* [61]. After nanofiltration, inhibition reached 9.8%, reflecting the concentration effect of pollutants, notably glyphosate and dissolved ions, in the retentate. This phenomenon is expected since nanofiltration acts as a selective barrier, concentrating compounds without degrading them, thus increasing their bioavailability and hence their toxic impact [62]. Electro-oxidation applied to the retentate results in a significant reduction in toxicity as inhibition drops to 3.7% with ${\mathrm{Ti}}_{4}O_{7}$ and 2.03% with BDD. This attenuation reflects the partial transformation of toxic compounds, such as glyphosate and AMPA, into more oxidized products such as ${\mathrm{CO}}_{2}$ the ${\mathrm{NO}}_{3}^{-}$ or the ${\mathrm{PO}}_{4}^{3-}$. These results underline the value of nanofiltration as a concentration step, but above all the effectiveness of its coupling with electro-oxidation to reduce the toxicity of the retentate, demonstrating a promising synergy for the treatment of contaminated water.

Although the BDD electrode presents slightly better efficiency for glyphosate degradation and mineralization, its main drawback lies in its high manufacturing cost, as its synthesis requires the use of high pressures and temperatures and expensive conductive substrates (silicon, niobium, or titanium) under complex technical conditions, which limits its large-scale application. By comparison ${\mathrm{Ti}}_{4}O_{7}$, a metal oxide of the Magnéli phase family is synthesized from ${\mathrm{TiO}}_{2}$, an inexpensive, abundant, and easily processed material [31]. In addition, ${\mathrm{Ti}}_{4}O_{7}$ offers good conductivity, satisfactory chemical stability, and, above all, a significant economic advantage. These characteristics make ${\mathrm{Ti}}_{4}O_{7}$ a promising alternative for drinking water and wastewater tertiary treatment applications, especially where cost-effectiveness is a key factor.

## 4. Materials and Methods

### 4.1. Materials

All chemicals used in this study were reagent grade or better and were used as received without further purification. Glyphosate (N-(phosphonomethyl) ($C_{3}H_{8}{\mathrm{NO}}_{5}P$, 98%), sodium sulfate (${\mathrm{Na}}_{2}{\mathrm{SO}}_{4}$), potassium sulfate ($K_{2}{\mathrm{SO}}_{4}$) and sulphuric acid ($H_{2}{\mathrm{SO}}_{4}$) used in the experiment were purchased from Sigma Aldrich (St. Louis, MO, USA). Aminomethylphosphonic acid (${\mathrm{CH}}_{6}{\mathrm{NO}}_{3}P$, 99%, AMPA), oxalic acid ($C_{2}H_{2}O_{4}$), oxamic acid ($C_{2}H_{3}{\mathrm{NO}}_{3}$), acetic acid ($C_{2}H_{4}O_{2}$), glyoxylic ($C_{2}H_{2}O_{3}$), glycoylic ($C_{2}H_{2}O_{3}$), formic (${\mathrm{CH}}_{2}O_{2}$) were obtained from Acros Organic (Geel, Belgium) and Alfa Aesar (Haverhill, MA, USA) analytical grade. Bioluminescent bacteria and LCK 487 LUMISTOX activation reagent were supplied by Hach Lange France SAS (Villeurbanne, France). The organic solvents and other chemicals used were HPLC or analytical quality from Sigma-Aldrich (St. Louis, MO, USA) and Merck (Darmstadt, Germany). Bacterial strain of *Vibrio fischeri* NRRL B-11177 involved in toxicity tests came from Hach Lange GmbH, Berlin, Germany. Osmotic adjusting solution (MilliQ water with 22% NaCl) and diluent (MilliQ water with 2% NaCl) were used for the preparation of bacterial solutions. Analytical standards were prepared in Milli-Q water (18 MΩ∙cm), and the glyphosate working solution was prepared in distilled water (15 MΩ∙cm).

### 4.2. Experimental Design

**Figure 15:**
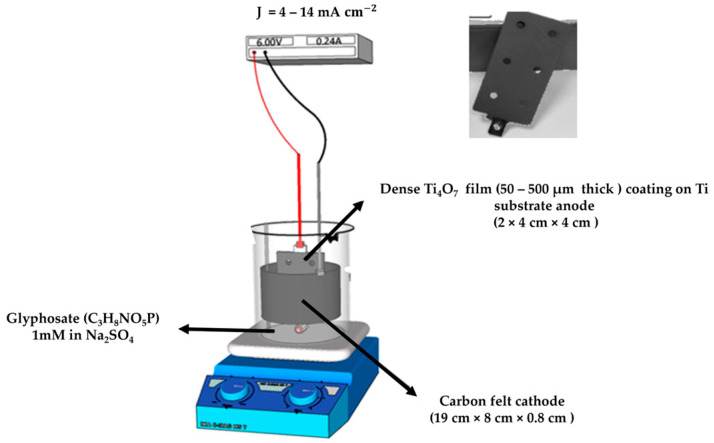
Schematic configuration.

All experiments were carried out in a 400 mL electrochemical cell (6 cm diameter), as depicted in Figure 15. A thin film of substoichiometric titanium oxide ${\mathrm{Ti}}_{4}O_{7}$ deposited on a Ti substrate with a total surface area of 32 ${\mathrm{cm}}^{2}$ (2 × 4 cm × 4 cm) and an electronic conductivity of 200 ${S\;\mathrm{cm}}^{-1}$ was used as the anode and placed in the center of the cell. The ${\mathrm{Ti}}_{4}O_{7}$ electrode was supplied by Saint-Gobain CREE, Cavaillon, France. The electrode was produced by plasma deposition of TiOx powder to form a thin film of 50–500 µm thick consisting mostly ${\mathrm{Ti}}_{4}O_{7}$ (particles size in the range of 20–60 µm) on a Ti substrate, as demonstrated by SEM and X-ray diffraction characterizations in our previous work [31]. A three-dimensional carbon felt (supplied by Alfa Aesar, 19 cm × 8 cm × 0.8 cm) was used as the cathode, covering the inner wall of the cell. A constant current was supplied by direct current (DC) Power Supply AL 781NX (Bürklin, Oberhaching, Germany) during the experiment. For each experiment, 200 mL of 1 mM aqueous glyphosate solution and 50 mM Na_2_SO_4_ (supporting electrolyte) were prepared. The solution was vigorously stirred by a magnetic bar during processing to enhance mass transport to the electrodes.

The electro-oxidation experiments were carried out at 20 °C for 8 h to study the effects of the main factors (pH and applied current density) on glyphosate degradation and mineralization in water. The effect of pH value was tested in the range 2 to 10 for different conditions in the presence of glyphosate 1 mM in ${\mathrm{Na}}_{2}{\mathrm{SO}}_{4}$: at pH 2 and 3 (addition of $H_{2}{\mathrm{SO}}_{4}$); at 3.61 (natural pH) and at pH 5; 7 and 10 (addition of NaOH). The current densities were varied from 4 to 14 ${\mathrm{mA}\;\mathrm{cm}}^{-2}$.

**Figure 16:**
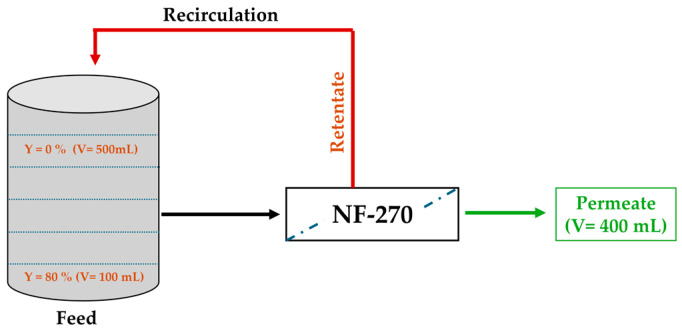
Diagram of NF device illustrating conversion rates (Y).

With the aim of coupling the nanofiltration (NF) process with the anodic electro-oxidation to study the degradation of synthetic glyphosate solution, an NF device (shown in Figure 16) with organic membranes of type NF-270 with an effective membrane area of 28 cm^2^ in a semi-open configuration was used. Prior to experimentation, the membrane was first soaked in ultrapure water to remove the preservative then compacted at 10 bars for at least one hour or until stable permeate flow was achieved. A synthetic ionic water from various simulated salts (Table 3) and containing glyphosate at 0.1 mM was prepared.

This model solution was designed to replicate the typical ionic composition of real effluents, particularly agricultural wastewater, while avoiding the variability and complexity inherent to natural environmental matrices. The use of this synthetic matrix enables the investigation of glyphosate degradation mechanisms in a controlled environment that is simplified yet representative of contaminated water conditions.

Then, 500 mL of the prepared synthetic solution were poured into the tank. Concentrates were returned to the tank and permeates collected up to a conversion ratio (Y) of 80% at a transmembrane pressure (TMP) of 10 bar. The experiment was repeated once in order to recuperate 200 mL of retentate.

### 4.3. Equipment and Analytical Procedures

Glyphosate concentrations (before and after electrochemical treatment) and degradation by-products (AMPA, glycine, and sarcosine) were monitored by HPLC-MS/MS equipped with a Luna 3 µm Polar Pesticides column (100 × 2.1 mm, Phenomenex, Torrance, CA, USA), protected by a Security Guard™ ULTRA guard (Phenomenex, Torrance, CA, USA); an LC-30AD pump (binary gradient mode) with the following mobile phases: 0.1% formic acid (A) and 0.1% acetonitrile (B). The flow rate was set to 0.3 ${\mathrm{mL}\;\min}^{-1}$, with an injection volume of 1 µL, and the column temperature was maintained at 15 °C. High-resolution electrospray ionization mass spectrometry was performed in MRM (Multiple Reaction Monitoring) mode in positive polarity. The detection conditions were as follows: desolvation line temperature of 200 °C, desolvation temperature of 450 °C, dry gas flow rate of 8${\;L\;\min}^{-1}$ and nitrogen as nebulization gases. The limit of quantification (LOQ) was 5 µM.

Inorganic ions (${\mathrm{PO}}_{4}^{3-}$, ${\mathrm{NH}}_{4}^{+}$ and ${\mathrm{NO}}_{3}^{-}$) generated during degradation of glyphosate were identified by an ion chromatography equipment. In the case of anion, Dionex ICS-6000 system was connected with an AS19 column (4 mm and 250 mm) and the eluent was KOH which was applied according to the elution gradient: 10 mM for 10 min, then gradient then up to 30 mM, and then 10 mM for 10 min (Thermo Fisher Scientific, Waltham, MA, USA). Dionex ICS-6000 was used to measure the cation concentration in the treated solution with the column CS12A and eluent of 20 mM methane sulfonic acid. Both columns were thermally controlled at 30 C, and conductivity detectors were used. Carboxylic acids were analyzed by Dionex ICS-6000 ion chromatography equipped with a BENSON BP-OA_2000 column and coupled to a UV detector selected at a wavelength of 210 nm. All samples were filtered through a 0.45 µm non-sterile nylon syringe filter (Branchia) prior to analysis.

Bacterial toxicity was assessed using the Microtox^®^ Model 500 analyzer (Modern Water Inc., York, UK), following the international standard ISO 11348-3 [63,64]. The toxic effect of glyphosate and its degradation products was determined by inhibiting the bioluminescence of the marine bacterium *Vibrio fischeri*. This light emission is directly linked to cellular respiration, allowing a direct correlation to be established with the metabolic activity of the cells [35]. A screening test of 10% was used to characterize inter-sample toxicity variability and identify the relative toxicity of each sample solution. The percentage inhibition of bacterial bioluminescence was calculated from the following Equation (7):

(7) $$I(t)\;=\;\frac{1-{\;\mathrm{LU}}_{\left(t\right)}}{R_{\left(t\right)}\;\times {\;\mathrm{LU}}_{\left(O\right)}}\;\times \;100$$

where LU_(t)_ is the intensity of luminescence emitted by the bacteria after contact t = 5 min or t = 15 min with the sample; and LU_(0)_ is the initial intensity of luminescence emitted by the bacteria before the addition of the sample.

Glyphosate mineralization into ${\mathrm{CO}}_{2}$, $H_{2}O$ and inorganic acids was determined by measuring TOC (Total Organic Carbon) concentrations during electrolysis using a Shimadzu TOC-VCPH analyzer (Shimadzu Scientific Instruments, Kyoto, Japan). The detection limit of the instrument is 0.5 μg $L^{-1}$, and the measuring principle is catalytic combustion at 680 °C (platinum catalyst). TOC removal efficiency corresponding to the mineralization rate was then determined from the following Equation (8) [51]:

(8) $$\%\;\mathrm{Mineralization}\;\mathrm{rate}\;=\;\frac{TOC_{0}-\;TOC_{t}}{T0C_{0}}\;\times \;100$$

where $TOC_{t}$ and $TOC_{0}$ are the experimental TOC concentrations at time t and initial time, respectively.

The mineralization current efficiency (MCE in %) was calculated from the following Equation (9):

(9) $$\mathrm{MCE}\;(\%)\;=\;\frac{n\;F\;V_{s\;}\left(TOC_{0}-\;TOC_{t}\right)}{4.32\;\times \;10^{7}m\;I\;t}\times \;100$$

where $TOC_{0}$ and $TOC_{t}$ are experimental initial TOC and TOC at time *t* (h) of treatment. *F* is the Faraday constant (96,487 ${C\;\mathrm{mol}}^{-1}$), $4.32\;\times \;10^{7}$ is a conversion factor (3600 ${s\;h}^{-1}$ × 12,000 mg of ${C\;\mathrm{mol}}^{-1}$), m is the number of carbon atoms of glyphosate (3C atoms), I is the applied current (A), $V_{s\;}$ is the total volume of solution, and *n* is the number of electron consumed to complete mineralize glyphosate to ${\mathrm{CO}}_{2}$, ${\mathrm{NO}}_{3}^{-}$ and ${\mathrm{PO}}_{4}^{3-}$ ions. *n* was determined based on the following reaction Equation (10):

(10) $$C_{3}H_{8}{\mathrm{NO}}_{5}P\;+\;8{\;H}_{2}O\;\;\to \;3{\mathrm{CO}}_{2}\;+\;{\mathrm{NO}}_{3}^{-}\;+\;{\mathrm{PO}}_{4}^{3-}\;+\;24{\;H}^{+}\;+\;20{\;e}^{-}$$

### 4.4. Specific Energy

Specific energy (*E_c_*), expressed in kilowatt-hours per gram of TOC removed (kWh ${g\;}^{-1}$ TOC), corresponds to the amount of electrical energy consumed to remove a unit mass of total organic carbon (TOC) during an electro-oxidation process. The evaluation of the energy efficiency of the process is relevant to compare different operating conditions or electrochemical configurations. It is calculated using the following expression:

(11) $$E_{c}\;=\;\frac{U\;I\;t}{\Delta TOC\;V\;1000}$$

where *I* is the applied current (A); *U* represents the cell voltage (V); *V* is the sample volume (L); Δ*TOC* = quantity of TOC eliminated (in ${g\;L}^{-1}$); *t* is the residence time (hours) and the factor 1/1000 converts watt-hours (Wh) to kilowatt-hours (kWh).

## 5. Conclusions

Electrochemical degradation of glyphosate using ${\mathrm{Ti}}_{4}O_{7}$ anodes (Magnéli phase) shows strong potential for water treatment, particularly under optimized acidic conditions (pH 3) and at high current densities (14 ${\mathrm{mA}\;\mathrm{cm}}^{-2}$), achieving 77.8% mineralization and complete glyphosate removal in 8 h. This efficiency is attributed to increased generation of hydroxyl radicals (^●^OH) and favorable adsorption of pollutants to the anode surface. However, the persistence of low amounts of recalcitrant carboxylic acids (acetic, oxamic) limits complete mineralization, underlining the need for prolonged treatments or hybrid processes. Crucially, toxicity tests confirmed detoxification, with *V. fischeri* inhibition dropping from 83.7% (maximum AMPA-related toxicity) to 2% after treatment, thus validating environmental safety.

Applied to nanofiltration retentate (glyphosate concentration 72.25 ${\mathrm{mg}\;L}^{-1}$), the comparison between anodes ${\mathrm{Ti}}_{4}O_{7}$ and BDD highlights strategic compromises that position the ${\mathrm{Ti}}_{4}O_{7}$ as a promising alternative:

Although BDD conducted to a slightly higher mineralization rate (90.5% vs. 81.3%), the difference remains moderate (9.2%), and the ${\mathrm{Ti}}_{4}O_{7}$ demonstrates solid performance in a matrix as complex as that produced by nanofiltration. The energy consumption observed for ${\mathrm{Ti}}_{4}O_{7}$ (6.09 kWh $g^{-1}$ TOC) remains close to that of BDD (5.48 kWh $g^{-1}$ TOC), indicating that the difference in energy efficiency does not constitute a major hindrance to its use. This slight overconsumption could be attributed to partial mineralization due to the persistence of certain organic intermediates and the complexity of the matrix, rather than to an intrinsic weakness of the anode.

Above all ${\mathrm{Ti}}_{4}O_{7}$ represents an economically advantageous and structurally stable solution, taking advantage of titanium dioxide’s abundance and ability to adapt to real-life effluents. Its performance, combined with its economic and environmental advantages, makes it a prime candidate for large-scale or decentralized applications.

The nanofiltration-electrochemical oxidation (NF-EO) combination is highly synergistic: nanofiltration concentrates glyphosate and reduces the volume to be treated, while electrochemical oxidation eliminates refractory pollutants. After treatment, residual toxicity drops to less than 5%, reflecting the elimination not only of glyphosate but also of its most toxic degradation by-product (AMPA). This performance enables us to comply with strict health standards for drinking water, such as the 0.7 ${µg\;L}^{-1}$ threshold set by the EPA for glyphosate or the WHO recommendations on micropollutants.

Future work should explore the integration of Fenton-based EAOP processes, in particular electro-Fenton, to optimize energy efficiency. Reduce the technical-economic gap between BDD and ${\mathrm{Ti}}_{4}O_{7}$ could revolutionize the management of water polluted by pesticides, in line with circular economy and sustainable development objectives.

## Figures and Tables

**Table 1:** Determination of apparent rate constants for different values of electrolysis current density under experimental conditions of Figure 2.

Electrode	$\mathrm{Current}\;\mathrm{Density}\;(mA\;cm^{-2})$	$k_{\mathrm{app}},\;\mathrm{Glyphosate}\;(10^{2}\;min^{-1})$	R^2^
Ti_4_O_7_	4	18.6 ± 0.7	0.98
	6	26.4 ± 1.1	0.98
	10	51.6 ± 5.7	0.90
	14	65.3 ± 6.2	0.94

**Table 2:** pH, conductivity and ionic composition of the synthetic ionic solution, the permeate and the retentate after NF.

Ions	Tank $(\mathrm{mg}\;L^{-1}$)	Permeate $(\mathrm{mg}\;L^{-1}$)	Retentate $(\mathrm{mg}\;L^{-1}$)
Phosphate $({\mathrm{PO}}_{4}^{3-}$)	9.3	0.0	15.6
Nitrate $({\mathrm{NO}}_{3}^{-}$)	7.3	3.3	10.6
Chlorure $({\mathrm{Cl}}^{-}$)	288.9	268.0	455.7
Sulfate $({\mathrm{SO}}_{4}^{2-}$)	54.2	7.5	459.7
Sodium $({\mathrm{Na}}^{+}$)	153.3	123.7	265.7
Ammonium $({\mathrm{NH}}_{4}^{+}$)	2.0	0.9	3.1
Potassium $(K^{+}$)	16.1	13.0	62.4
Magnesium $({\mathrm{Mg}}^{2+}$)	21.0	7.2	62.2
Calcium $({\mathrm{Ca}}^{2+}$)	50.5	20.1	141.4
Glyphosate	17.0	0.0	72.3
TOC	5.4	1.2	20.7
pH	7.49	7.63	8.45
Conductivity (µS ${\mathrm{cm}}^{-1})$	1480	1000	2380

**Table 3:** Ionic composition of the prepared synthetic solution.

Compounds	$\mathrm{Concentrations}\;(mg\;L^{-1})$
NaCl	200
${\mathrm{CaCl}}_{2}\cdot 2H_{2}O$	238.5
${\mathrm{MgCl}}_{2}\cdot 6H_{2}O$	200
Na_2_HPO_4_·2H_2_O	9
${\mathrm{Na}}_{2}{\mathrm{SO}}_{4}$	75
${\mathrm{NaHCO}}_{3}$	200
KCl	30
${\mathrm{NaNO}}_{3}$	5
${\mathrm{NH}}_{4}\mathrm{Cl}$	2

## Data Availability

The original contributions presented in this study are included in the article. Further inquiries can be directed to the corresponding authors.
